# Impact of the COVID-19 Pandemic on Partner Relationships and Sexual and Reproductive Health: Cross-Sectional, Online Survey Study

**DOI:** 10.2196/20961

**Published:** 2020-08-06

**Authors:** Guanjian Li, Dongdong Tang, Bing Song, Chao Wang, Shen Qunshan, Chuan Xu, Hao Geng, Huan Wu, Xiaojin He, Yunxia Cao

**Affiliations:** 1 Reproductive Medicine Center, Department of Obstetrics and Gynecology The First Affiliated Hospital of Anhui Medical University National Health Commission Key Laboratory of Study on Abnormal Gametes and Reproductive Tract Hefei China; 2 Key Laboratory of Population Health Across Life Cycle, Ministry of Education of the People’s Republic of China Anhui Province Key Laboratory of Reproductive Health and Genetics Hefei China

**Keywords:** COVID-19, survey, novel coronavirus, sexual behavior, sexual health, reproductive health, young adults, youth, China

## Abstract

**Background:**

In the past few months, the coronavirus disease (COVID-19) pandemic has caused extensive economic and social damage.

**Objective:**

The purpose of this study was to assess the impact of COVID-19–related measures on partner relationships and sexual and reproductive health in China.

**Methods:**

From May 1 to 5, 2020, 3500 young Chinese individuals were recruited through WeChat or Weibo to participate in a survey to obtain information on sexual and reproductive health (eg, sexual desire, frequency of sexual intercourse, sexual satisfaction, etc). The questionnaire also collected demographic data (eg, age, race, education, current financial status, sexual orientation, relationship status, etc).

**Results:**

In total, 967 participants were included in the sexual health analysis. Due to the COVID-19 pandemic and related containment measures, 22% of participants (n=212) reported a decrease in sexual desire; 41% (n=396) experienced a decrease in the sexual intercourse frequency; 30% (n=291) reported an increase in the frequency of masturbation; 20% (n=192) reported a decrease in alcohol consumption before or during sexual activities, and 31% (n=298) reported a deterioration in partner relationships during the pandemic. The logistic regression analysis indicated that the following influenced partner relationships: accommodations during the pandemic (*P*=.046; odds ratio [OR] 0.59; 95% CI 0.30-0.86); exclusive relationship status (yes or no) (*P*<.001; OR 0.44; 95 % CI 0.27-0.73); sexual desire (*P*=.02; OR 2.01; 95% CI 1.38-2.97); and sexual satisfaction (*P*<.001; OR 1.92; 95% CI 1.54-2.50). COVID-19 also caused disruptions in reproductive health services such as prenatal and postnatal care, childbirth and abortion services, contraception availability, and the management of sexually transmitted infections.

**Conclusions:**

Our results show that many young people have wide-ranging issues affecting their sexual and reproductive health due to the COVID-19 pandemic and related containment measures. Strategies and guidelines are needed to safeguard the sexual and reproductive health of young people during this pandemic.

## Introduction

In the past few months, the coronavirus disease (COVID-19) pandemic and related containment measures have caused extensive economic and social damage to many countries like China and the United States [1-3].

From February to April 2020, all schools and many businesses in China were closed. Everyone was required to implement “social distancing,” and the government restricted travel, parties, and outdoor activities [4,5]. In addition, many provinces have implemented guidelines to reduce pressure on the health care system, including the suspension of nonemergency medical care and elective surgeries [6]. These disruptions have had a significant impact on the physical and mental health, as well as quality of life, of individuals [7,8].

So far, there is little report about the effects of COVID-19 on sexual and reproductive health [9,10]. Sexually active young people are facing more and more health challenges globally [11,12], and various aspects of their reproductive and sexual health may be affected by COVID-19. On the one hand, many young people are facing economic and psychological pressures caused by job loss or suspension of schooling. On the other hand, separation from sexual partners and lack of access to comprehensive health care services may be increasing the risk of experiencing negative sexual health outcomes.

Network-based sexual health risk assessments have been considered acceptable, and internet technology has become a powerful tool to promote health [13,14]. To assess the impact of COVID-19 pandemic and related containment measures on partner relationships and sexual and reproductive health, we conducted a series of preliminary analyses using data from an internet-based survey among Chinese youths and young adults.

## Methods

### Participant Recruitment

We conducted a cross-sectional, online survey using Questionnaire Star, an online questionnaire survey platform with 82,000,000 users in China. A 20-item survey (Multimedia Appendix 1) was constructed to assess changes in people's sexual and reproductive health during the COVID-19 pandemic. Between May 1 and 5, 2020, 3500 participants in China received links and emails from WeChat or Weibo (similar to WhatsApp and Twitter, respectively), inviting them to participate in a confidential, 20-minute, online survey about sexual and reproductive health on the Questionnaire Star platform.

### Data Collection

Multiple reminders were sent via messaging software, and the invitation letter stated that the current survey was for participants who were sexually active. Approximately US $5 was offered as an incentive for participants who completed the questionnaire. Duplicate entries were prevented by restricting users with the same IP (Internet Protocol) address from accessing the survey more than once. A missed answer reminder component prompted participants about unfinished questionnaires in real time, and incomplete questionnaires were not submitted to the system. In addition, participants were unable to submit the questionnaire if their total answering time was less than 3 minutes.

Before entering the online survey system, all participants reviewed and approved the electronic consent page. This research was approved by the Ethics Review Committee of Anhui Medical University for research and publication purposes.

The questionnaire collected data on age, race, education, current financial status, SARS-CoV-2 (severe acute respiratory syndrome coronavirus 2) infection status of participants and those around them, self-rated health, accommodations before and during the pandemic, medical and surgical history of participants and their partners, pregnancy (if female), abortion history, sexual orientation, relationship status (ie, exclusive partnership or not), sexual desire, frequency of sexual intercourse, sexual satisfaction, alcohol consumption before or during sexual activities, frequency of masturbation, frequency of use of pornographic content, proportion of condom use (in sexual encounters), risky sexual behavior, and presence of sexually transmitted infections (STIs). Symptoms were assessed for during the COVID-19 pandemic. Before the questionnaire was distributed, 10 college students and 2 professors participated in a pilot study to verify and modify the contents of the questionnaire.

Participants were included in the current analysis if they were 15-35 years old, live in China, and reported penetrative sex (defined as insertion of penis into vaginal or anal orifices) at least once at any time in the past 6 months (n=1076). Of those invited, 35.7% (n=1249) of individuals completed the survey. Foreigners (n=11); homosexual or bisexual individuals (n=16); people, or people with partners, who had COVID-19 (n=0) or had contact with someone with COVID-19 (n=2); and those with systemic diseases (n=23) and other serious conditions that can cause sexual dysfunction (n=10) were excluded since their experiences may differ, and their representation in the sample was small.

Due to the complexity of physiological conditions and interference factors, people with STIs (n=8) and those who were pregnant (n=25) or had undergone a recent abortion (n=6) were also excluded from the sexual health analysis, but the COVID-19–related impact on several aspects of their reproductive health and rights were measured. Participants who were pregnant were asked “Have you experienced any difficulties in obtaining maternal care or delivery services due to COVID-19 or the plans to manage it?”. Participants who reported a recent abortion were asked “Have you experienced any difficulties in obtaining abortion or post-abortion care due to COVID-19 or the plans to manage it?”. Participants who reported STIs were asked “Have you experienced any difficulties in obtaining medical advice or management due to COVID-19 or the plans to manage it?”. All participants who answered “yes” were invited to provide a description of their difficulties. Apart from that, all participants were asked to fill in a response to “Have you experienced a shortage of contraceptives during the pandemic?”.

## Results

### User Statistics

A total of 967 participants were included in the sexual health analysis. The characteristics of the study participants are shown in Table 1. The mean age was 26.6 (SD 4.86) years (range 16 to 35 years), and 55.9% (n=541) were male. All participants were Han Chinese. Of the 967 participants, almost half (n=416, 43%) reported a recent deterioration in their financial situation, and 8% (n=75) reported a poor state of health.

**Table 1:** Participants’ demographic characteristics (N=967).

Characteristic		Total (N=967), n (%)	Male (n=541), n (%)	Female (n=426), n (%)	*F*	*P* value
**Age (year)**					2.26	.13
	15-25	389 (40)	229 (43)	160 (36)		
	25-35	578 (60)	312 (57)	266 (64)		
**Education level**					3.10	.21
	College or below	491 (47)	263 (45)	228 (50)		
	Bachelor	405 (42)	240 (44)	165 (40)		
	Master or above	71 (10)	38 (11)	33 (10)		
**Current financial situation**					21.15	<.001
	Fine	221 (23)	134 (25)	87 (20)		
	Unchanged	330 (34)	151 (28)	179 (42)		
	Deteriorated	416 (43)	256 (47)	160 (38)		
**Self-rated health**					2.76	.25
	Fine	282 (29)	154 (28)	128 (30)		
	General	610 (63)	351 (65)	259 (61)		
	Poor	75 (8)	36 (7)	39 (9)		
**Accommodation** **(before pandemic)**					0.51	.77
	Campus dormitory	416 (43)	229 (42)	187 (44)		
	House with parents	348 (36)	200 (37)	148 (35)		
	House without parents	203 (21)	112 (21)	91 (21)		
**Accommodation (during pandemic)**					1.37	.24
	House with parents	706 (73)	403 (74)	303 (71)		
	House without parents	261 (27)	138 (26)	123 (29)		

### COVID-19–Related Impact on Sexual Health

COVID-19–related impact on sexual health is summarized in Table 2. In all, 68.8% (n=665) of students included in the analysis reported that they were currently in an exclusive relationship. There were significant differences in sexual health and outcomes between students in an exclusive relationship compared to those who were not in an exclusive relationship.

Of the 967 participants included in the analysis, 22% (n=212) reported a decrease in sexual desire, 41% (n=396) experienced a decrease in the frequency of sex, 20% (n=192) reported a recent decrease in alcohol consumption before or during sexual activities, and 10% (n=94) reported a decrease in risky sexual behavior. In addition, 31% (n=298) reported partner relationship deterioration during the pandemic (Table 2). With regard to the frequency of masturbation, 30% (n=291) of participants reported an increase in masturbation during the pandemic, while 23% (n=227) reported an increase in the use of pornography.

The logistic regression analysis indicated that accommodations during the pandemic, exclusive relationship status, sexual desire, and sexual satisfaction were closely related to partner relationships (Table 3).

**Table 2:** Coronavirus disease (COVID-19)–related impact on sexual health (N=967).

Items		Total (N=967)	In an exclusive relationship (n=665), n (%)	Not in an exclusive relationship^a^ (n=302), n (%)	*F*	*P* value
**Partner relationship**					40.76	<.001
	Fine	205 (21)	133 (20)	72 (24)		
	General	464 (48)	285 (43)	179 (59)		
	Deteriorated	298 (31)	247(37)	51 (17)		
**Sexual desire**					42.52	<.001
	Fine	126 (13)	86 (13)	40 (13)		
	General	629 (65)	395 (59)	234 (77)		
	Deteriorated	212 (22)	184 (28)	28 (09)		
**Sexual** **frequency**					153.38	<.001
	Increased	223 (23)	175 (26)	48 (16)		
	Unchanged	348 (36)	304 (46)	44 (15)		
	Decreased	396 (41)	186 (28)	210 (70)		
**Sexual satisfaction**					33.19	<.001
	Increased	115 (12)	54 (8)	61 (20)		
	Unchanged	709 (73)	498 (75)	211 (70)		
	Decreased	143 (15)	113 (17)	30 (10)		
**Consumed alcohol before or during sexual activities**					132.01	<.001
	Increased	58 (6)	44 (7)	14 (5)		
	Unchanged	717 (74)	555 (83)	162 (54)		
	Decreased	192 (20)	66 (10)	126 (42)		
**Frequency of masturbation**					21.99	<.001
	Increased	291 (30)	189 (28)	102 (34)		
	Unchanged	261 (27)	206 (31)	55 (18)		
	Decreased	106 (11)	78 (12)	28 (9)		
	None	309 (32)	192 (29)	117 (39)		
**Frequency** **of pornography use**					30.71	<.001
	Increased	227 (23)	123 (19)	104 (34)		
	Unchanged	330 (34)	242 (36)	88 (29)		
	Decreased	115 (12)	89 (13)	26 (9)		
	None	295 (31)	211 (32)	84 (28)		
**Proportion** **of condom use**					5.25	.07
	Increased	97 (10)	61 (9)	36 (12)		
	Unchanged	735 (76)	501 (75)	234 (77)		
	Decreased	135 (14)	103 (16)	32 (11)		
**Risky sexual behaviors**					18.87^b^	<.001
	Increased	4 (1)	3 (1)	1 (1)		
	Unchanged	76 (8)	55 (8)	21 (7)		
	Decreased	94 (010)	46 (7)	48 (16)		
	None	793 (82)	561 (84)	232 (77)		

^a^Chi-square test was performed between the exclusive relationship group and nonexclusive relationship group.

^b^The “increased” data were merged with the “unchanged” data.

**Table 3:** Risk factors related to partner relationships determined by the logistic regression analysis.

Variable	Univariate analysis	Multivariate analysis		
	*P* value	OR^a^	*P* value	95% CI
Age (year)	.03	1.38	.41	1.13-1.80
Education level	.66	—^b^	—	—
Current financial situation	.16	—	—	—
Self-rated health	.02	1.85	.19	1.37-2.63
Accommodations (before pandemic)	.21	—	—	—
Accommodations (during pandemic)	.02	0.59	.046	0.30-0.86
In/not in an exclusive relationship	<.001	0.44	<.001	0.27-0.73
Sexual desire	.002	2.01	.02	1.38-2.97
Sexual satisfaction	<.001	1.92	<.001	1.54-2.50
Alcohol consumption before or during sexual activities	.36	—	—	—
Frequency of masturbation	.005	0.63	.21	0.46-0.88
Frequency of pornography use	.29	—	—	—
Proportion of condom use	.52	—	—	—
Risky sexual behaviors	.41	—	—	—

^a^OR: odds ratio.

^b^Not applicable.

### COVID-19–Related Impact on Reproductive Health

**Figure 1:**
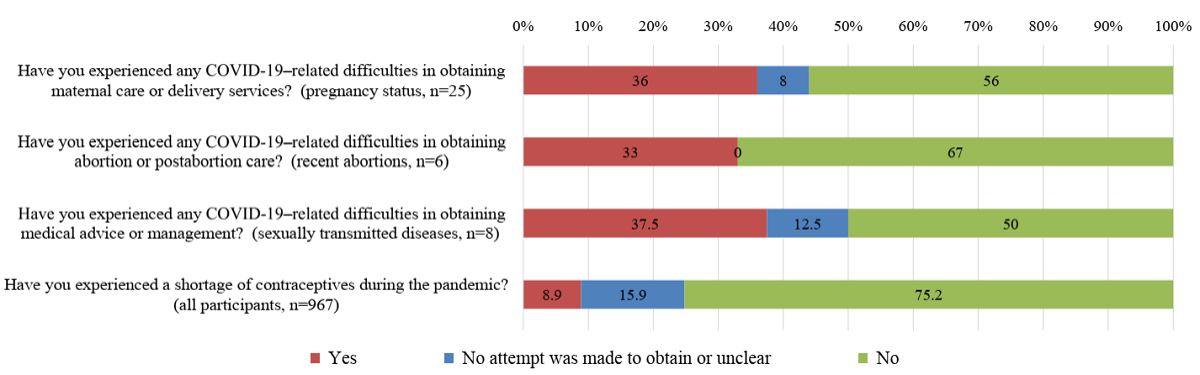
Self-reported coronavirus disease (COVID-19)–related impact among respondents.

COVID-19–related impact on reproductive health is shown in Figure 1. Nine participants who were pregnant reported having difficulties accessing maternal care or delivery services due to COVID-19 or related measures. These difficulties included shortages in the number of hospital beds available for childbirth, restrictions on the number of people accompanying pregnant women for examination or hospitalization, and failure to receive timely prenatal examination.

Participants who reported a recent abortion described difficulties primarily with making appointments to see a doctor or for surgeries. Three participants who reported STIs experienced difficulties with medical management, mainly booking a doctor’s appointment and accessing medicines such as antibiotics.

In the current study, the proportion of condom usage was unchanged due to COVID-19. However, 8.9% (n=86) of participants said they had experienced a shortage of contraceptives.

## Discussion

### Principal Findings

Our study provides preliminary evidence on the direct impact of the COVID-19 pandemic and related containment measures on partner relationships and sexual and reproductive health. The results show that many young people had decreased sexual desire and frequency of sexual intercourse due to COVID-19. In addition, a relatively large number of participants reported a significant reduction in alcohol-related sexual consequences and risky sexual behavior. Increased family supervision or interference, less personal freedom overall, and poor mental health and partner relationships are likely contributors to these changes in sexual behavior.

We found that many participants reported an increase in masturbation frequency and use of pornography. Although masturbation may have helped some people achieve sexual satisfaction without the risk of SARS-CoV-2 infection, a high masturbation rate is related to a decrease in quality of life and sexual satisfaction [15]. High-frequency pornography use may also negatively impact sexual function and quality of life [16].

Our research also provides preliminary evidence of interruptions in reproductive health services due to COVID-19, such as prenatal and postnatal examination, delivery and abortion services, contraception availability, and STI management. In addition, we found that even in a country with a sound drug supply system such as China, contraceptives in some areas were out of stock or in short supply during the pandemic.

### Limitations

The limitations of the current study include the use of a self-designed questionnaire and the reliance upon self-reporting in the midst of the constantly changing prevalence of COVID-19. As a result, our findings are based on cross-sectional data from local convenient samples. In addition, the impact of the COVID-19 pandemic on the sexual health of special groups, including lesbian, gay, bisexual, and transgender people, and people living with HIV, has not been reported in the current research. Further large-scale longitudinal studies are needed to understand the impact of the pandemic on sexual and reproductive health in different regions and populations.

### Conclusions

The COVID-19 pandemic and related containment measures affect young people's sexual health, and targeted interventions are needed to improve health and well-being. In addition, since society is currently focusing on COVID-19 response, basic reproductive health services and supply chain operations have been disrupted. Such services should be protected from disruption and be delivered during the pandemic.

## Appendix

Multimedia Appendix 1 Questionnaire (English version).

## Abbreviations

COVID-19: coronavirus disease
IP: Internet Protocol
SARS-CoV-2: severe acute respiratory syndrome coronavirus 2
STI: sexually transmitted infection
