# Supplementary material for: Impact of the COVID-19 Pandemic on Partner Relationships and Sexual and Reproductive Health: Cross-Sectional, Online Survey Study
Source: J Med Internet Res. 2020 Aug 6;22(8):e20961. doi: 10.2196/20961 (PMC7419154; doi:10.2196/20961)
Supplement: Multimedia Appendix 1 [file jmir_v22i8e20961_app1.docx]

**Multimedia Appendix 1**

（The English version of the questionnaire）

Your gender:

Your age:

Your ethnicity:

1. What is your education level?

A. college or below

B. bachelor

C. master or above

2. What was your current financial situation during the COVID-19 pandemic?

1. Improved
2. Unchanged
3. Deteriorate

3. In general, would you say your health is?

A. fine

B. general

C. poor

4. (1) Have you or your partner ever had a COVID-19 disease?

A. Yes

B. No

4. (2) Have you or your partner ever had contact with a person with a COVID-19 disease?

A. Yes

B. No

5. What was your accommodation type before the COVID-19 pandemic?

1. campus dormitory
2. house with parents
3. house without parents

6. What was your accommodation type during the COVID-19 pandemic?

1. house with parents
2. house without parents

7. Have you had sex in the past six months?

1. Yes
2. No

8. Are you currently in an exclusive relationship where you are emotionally involved during the COVID-19 pandemic?

1. Yes
2. No

9. What is your sexual orientation?

1. Heterosexual
2. Bisexual
3. Homosexual
4. Asexual

10. Are you in the following state (multiple choice)

1. Serious systemic diseases (Respiratory system, digestive system, circulatory system, nervous system, motor system, urogenital system, endocrine system, immune system, cancer, Mental disorders, etc)
2. Sexually transmitted disease (Syphilis, AIDS, condyloma, neisseria gonorrhoeae, chlamydial trachomatis genital infection, nongonococcal urethritis, others)
3. Other serious conditions that lead to sexual dysfunction
4. Pregnant or recent childbirth
5. Recent abortion

F. Any important medical histories and surgical histories that need to be disclosed (detail information)

11. How about your partner relationship during the COVID-19 pandemic?

1. Fine
2. General
3. Deteriorate

12. How about your sexual desire during the COVID-19 pandemic?

1. Increased
2. Reduced
3. Unchanged

13. How about the frequency of your sexual behavior during the COVID-19 pandemic?

1. Increased
2. Reduced
3. Unchanged

14. How about your sexual satisfaction during the COVID-19 pandemic?

1. Increased
2. Reduced
3. Unchanged

15. How about your consumed alcohol before or during sexual activities during the COVID-19 pandemic?

1. Increased
2. Reduced
3. Unchanged

16. How did your masturbation frequency change during the pandemic?

1. Increased
2. Reduced
3. Unchanged
4. Never had

17. During the COVID-19 pandemic, how did the frequency of your pornography use change?

1. Increased
2. Reduced
3. Unchanged
4. Never had

18. How did your condom use proportion (in sexual encounters) change during the pandemic?

1. Increased
2. Reduced
3. Unchanged

19. How is your risky sexual behaviors (defined as inconsistent condom use, "casual" sexual partnerships, or multiple sexual partnerships) during the COVID-19 pandemic?

1. Increased
2. Reduced
3. Unchanged
4. Never had

20. Have you experienced a shortage of contraceptives during the pandemic?

A. Yes

B. No

(Optional questions, for pregnancy or recent childbirth) Have you experienced any difficulties in obtaining maternal care or delivery services due to COVID-19 or the plans to manage it?

1. Yes

( Detail information: )

1. No

(Optional questions, for recent abortion) Have you experienced any difficulties in obtaining abortion or post-abortion care due to COVID-19 or the plans to manage it?

1. Yes

( Detail information: )

1. No

(Optional questions, for who have sexually transmitted diseases) Do you experienced any difficulties in obtaining medical advice or management due to COVID-19 or the plans to manage it?

1. Yes

( Detail information: )

1. No
